# Identification of positron emission tomography (PET) tracer candidates by prediction of the target-bound fraction in the brain

**DOI:** 10.1186/s13550-014-0050-6

**Published:** 2014-09-23

**Authors:** Markus Fridén, Marie Wennerberg, Madeleine Antonsson, Maria Sandberg-Ställ, Lars Farde, Magnus Schou

**Affiliations:** Respiratory Inflammation and Autoimmunity Innovative Medicines, AstraZeneca R&D, Mölndal, Sweden; Translational PKPD, Department of Pharmaceutical Biosciences, Uppsala University, Uppsala, Sweden; Cardiovascular and Metabolic Diseases Innovative Medicines, AstraZeneca R&D, Mölndal, Sweden; CNS & Pain Innovative Medicines, AstraZeneca R&D, Södertälje, Sweden; AstraZeneca Translational Science Centre, Department of Clinical Neuroscience, Karolinska Institutet, Stockholm, Sweden

**Keywords:** Positron emission tomography, Non-specific binding, Imaging, Receptor occupancy

## Abstract

**Background:**

Development of tracers for imaging with positron emission tomography (PET) is often a time-consuming process associated with considerable attrition. In an effort to simplify this process, we herein propose a mechanistically integrated approach for the selection of tracer candidates based on *in vitro* measurements of ligand affinity (K_d_), non-specific binding in brain tissue (V_u,brain_), and target protein expression (B_max_).

**Methods:**

A dataset of 35 functional and 12 non-functional central nervous system (CNS) PET tracers was compiled. Data was identified in literature for K_d_ and B_max_, whereas a brain slice methodology was used to determine values for V_u,brain_. A mathematical prediction model for the target-bound fraction of tracer in the brain (f_tb_) was derived and evaluated with respect to how well it predicts tracer functionality compared to traditional PET tracer candidate selection criteria.

**Results:**

The methodology correctly classified 31/35 functioning and 12/12 non-functioning tracers. This predictivity was superior to traditional classification criteria or combinations thereof.

**Conclusions:**

The presented CNS PET tracer identification approach is rapid and accurate and is expected to facilitate the development of novel PET tracers for the molecular imaging community.

**Electronic supplementary material:**

The online version of this article (doi:10.1186/s13550-014-0050-6) contains supplementary material, which is available to authorized users.

## Background

Positron emission tomography (PET) is a molecular imaging technique that is being increasingly used in medical research and drug development. The non-invasive nature of PET, the low chemical mass of the radiolabeled probe used in the emission measurement (usually only micrograms), and the relatively low radiation burden associated with a PET measurement have positioned PET as one of the key enabling technologies in translational medicine. PET can be applied for a wide range of purposes, but all are crucially dependent on the availability of suitable radiotracers for the emission measurements.

**Figure 1:**
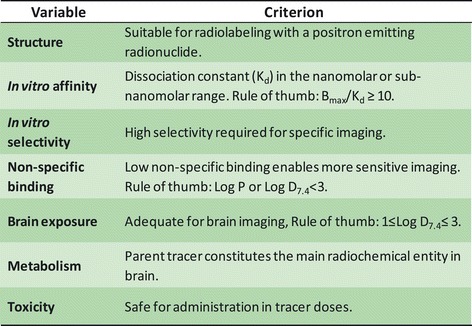
**Commonly applied criteria for CNS candidate tracer selection.**

The development of PET tracers for the central nervous system (CNS) is often a time-consuming process associated with considerable attrition. Thus, despite the plethora of novel targets of interest for PET imaging, the availability of useful tracers constitutes a bottleneck in nuclear medicine and drug industry. The high attrition rate in tracer development can be attributed to the many properties that a successful CNS tracer has to satisfy including tracer affinity, non-specific binding, blood–brain barrier transport, metabolic stability, etc. (Figure 1) [1-5].

Over the years, considerable efforts have been directed to the development of methods for selection of CNS PET tracer candidates. In particular, the prediction of non-specific brain tissue binding has been in focus, for which *in silico*, *in vitro*, or bio-mathematical methods have been applied [6-9]. Recently, a selection method comprising the composite of weighted physicochemical parameters (CNS PET multiparameter optimization or ‘MPO’), free fractions in plasma and the brain, as well as membrane permeability has been reported [10].

The aim of the present work was to develop and examine an integrated model for identification of promising CNS tracer candidates. The model includes only estimates of non-specific binding, tracer affinity, and target protein expression in the brain. The outcome parameter is the target-bound fraction of tracer in the brain (f_tb_). The model was validated on a set of 47 successful or failed tracer developments.

## Methods

### CNS PET tracer dataset

A CNS PET tracer dataset was generated by compilation of 31 PET tracers that either have been evaluated in house at the PET centre at Karolinska Institutet, Sweden, or are related to targets that have been examined at the PET centre. Complementing this dataset, a subset of 18 tracers with measured unbound fraction in brain homogenate was included from a recently published CNS PET tracer database [10]. Tracers were classified as functional or non-functional based on their utility *in vivo* for reliable quantification of specific target binding. Two tracer molecules were excluded from the dataset: [^11^C]GSK215083 due to insufficient selectivity and [^11^C]RWAY due to radioactive metabolites that potentially confounded PET images. The final dataset comprised 35 validated functioning PET tracers and 12 non-functioning tracers (Table 1).

**Table 1:** **CNS PET tracer dataset** ^**a**^

		**Target**	***In vitro*** **data**				**PET data**	
			**B**_**max**_	**K**_**d**_	**V**_**u,brain**_	**f**_**tb**_	**BP**_**ND**_	**f**_**tb**_
			**(nM)** ^**b**^	**(nM)**	**(mL/g brain)**			
[^18^ F]2-FA-85380	Yes	nAChr a4b2	0.7	0.145	1.7	0.74	1.8	0.64
[^11^C]AFM	Yes	SERT	38	1.04	46	0.44	1.4	0.58
[^18^ F]Altanserin	Yes	5HT2a	89	0.32	122	0.70	1.06	0.51
[^11^C]AZ10419369	Yes	5HT1b	9.8^c^	0.37	30	0.47	1.3	0.57
[^11^C]AZD2184	Yes	Amyloid	1,407	4.9	33	0.90	1.1	0.52
[^11^C]AZD2995	Yes	Amyloid	1,407	6.2	7	0.97	0.6	0.38
[^18^ F]AZD4694	Yes	Amyloid	1,407	2.3	205	0.75	1.2	0.55
[^11^C]CP-126998	Yes	AchE	211	0.48	41	0.92		
[^11^C]DASB	Yes	SERT	38	3.5	31	0.26	1.6	0.62
[^18^ F]Fallypride^f^	Yes	D2	27	0.03	18	0.98	22.2	0.96
[^18^ F]Fallypride^g^	Yes	D2	0.9	0.03	18	0.63	2.11	0.68
[^18^ F]FE-PE2I	Yes	DAT	212	12	62	0.22	4.1	0.80
[^18^ F]FEPPA[iv]	Yes	TSPO	58	0.07	15	0.98	4.4	0.81
[^11^C]FLB457	Yes	D2	0.9	0.02	26	0.63	2.6	0.72
[^11^C]Flumazenil	Yes	GABA	71	0.7	3.2	0.97	5.8	0.85
[^18^ F]FP-CIT	Yes	DAT	212	33	36	0.15	1.0	0.50
[^11^C]GR103545	Yes	KOR	3.75^c^	0.048	41	0.66	2.18	0.69
[^11^C]GR205171	Yes	NK1	55	0.016	57	0.98	14.5	0.94
[^11^C]GSK189254A	Yes	H3	8.4	0.08	8.5	0.93	1.3	0.57
[^11^C]Harmine	Yes	MAO-A	270	5	25	0.68	1.7	0.63
[^11^C]MADAM	Yes	SERT	38	0.06	90	0.88	1.4	0.58
[^11^C]McN5652	Yes	SERT	38	0.2	238	0.44	0.50	0.33
[^11^C]MDL100907	Yes	5HT2a	89	0.24	17	0.96	1.3	0.57
[^11^C]MePPEP	Yes	CB1r	47	0.1	296	0.61	5.5	0.85
[^18^ F]MPPF	Yes	5HT1a	350	3.3	14	0.89	1.6	0.62
[^11^C]NNC112	Yes	D1	93	0.18	70	0.88	2.85	0.74
[^11^C]PBR28	Yes	TSPO	58	1.8	11	0.75	3.99	0.80
[^11^C]PE2I	Yes	DAT	212	4.9	39	0.53	8.0	0.89
[^11^C]PHNO	Yes	D2/D3	26.5^d^	0.56	11	0.81	2.5	0.71
[^11^C]PIB	Yes	Amyloid	1,407	2.5	250	0.69	0.85	0.46
[^11^C]PK11195	Yes	TSPO	58	4.3	59	0.19	0.18	0.15
[^11^C]Raclopride	Yes	D2	27	2.5	9.4	0.53	2.6	0.72
[^18^ F]Spiperone	Yes	D2	27	0.028	147	0.87		
[^11^C]SB207145	Yes	5HT4	21	0.037	4.4	0.99	3.4	0.77
[^11^C]SCH23390	Yes	D1	93	2.1	32	0.58	1.8	0.64
[^11^C]WAY100635	Yes	5HT1a	350	1.1	14	0.96	7.4	0.88
[^11^C]Citalopram	No	SERT	38	4.8	60	0.12	0.1	0.09
[^11^C]Clomipramine	No	SERT	38	0.15	863	0.23	0.1	0.09
[^11^C]CPEB[iv]	No	ORL-1	13.5^e^	1.1	143	0.08	0.1	0.09
[^11^C]Desipramine	No	NET	5	0.63	264	0.03	0.1	0.09
[^11^C]Diazepam	No	GABA	71	7	20	0.34	0.1	0.09
[^11^C]MeNER	No	NET	5^c^	2.5	31	0.06	0.3	0.23
[^11^C]NE100	No	Sigma	23^e^	1.2	96	0.17	0.1	0.09
[^11^C]Nisoxetine	No	NET	5	0.73	58	0.11	0.1	0.09
[^18^ F]Paroxetine	No	SERT	38	0.065	876	0.40	0.1	0.09
[^11^C]Remoxipride	No	D2	27	270	6.3	0.02	0.1	0.09
[^11^C]Sertraline	No	SERT	38	0.15	4,184	0.06	0.1	0.09
[^11^C]Venlafaxine	No	SERT	38	7.5	10	0.33	0.1	0.09

^a^An extended version of this table is provided as supporting information (Additional file 1: Table S1), which includes literature references to B_max_, K_d_, and BP_ND_ for each tracer, details of V_u,brain_ determination, calculated molecular descriptors and CNS PET MPO score, and the region of brain tissue interest.

^b^Data refers to human brain tissue unless otherwise specified.

^c^Monkey.

^d^Dog.

^e^Rat.

^f^B_max_ value refers to caudate.

^g^B_max_ value refers to thalamus.

For each tracer, target density (B_max_), the affinity (K_d_) of the tracer for the target, and the non-displaceable binding potential (BP_ND_) were obtained from the literature. A single value of K_d_ was entered into the database even if more than one value has been reported in literature. Selection preference was given to (1) reports containing data from human material, (2) reports containing data on both K_d_, B_max_, or BP_ND_, or (3) the first encountered report.

The unbound volume of distribution in the brain (V_u,brain_) describing the extent of non-specific partitioning was determined for 31 tracers using a previously described high-throughput brain slice method [11]. Compound material was not available for 16 tracers and V_u,brain_ was instead calculated from reported measurements of unbound fraction in homogenized brain tissue and the tracer pK_a_ using a pH-partition model [12]. Molecular descriptors including ClogP, ACDlogD7.4, polar surface area (PSA), molecular weight (MW), hydrogen bond donor count (HBD), and ACDpK_a_ were calculated and used to generate the CNS PET multiparameter optimization (MPO) score [10]. An extended version of Table 1 with complete literature references is provided as supplementary material and includes the calculated molecular properties (Additional file 1).

### Equations and relationships

A mathematical relationship for f_tb_ was derived from a model of the total concentration of tracer in brain tissue (C_brain_, pmol/g brain) comprising non-specific tracer and target-bound tracer. The concentration of non-specific tracer is determined by the product of V_u,brain_ (mL/g brain) and the unbound tracer concentration in the brain interstitial fluid (C_u,brainISF_, nmol/L ISF). The concentration of target-bound tracer is described by a non-linear expression with C_u,brainISF_, K_d_ (nmol/L), and B_max_ (nmol/g brain) (Eq. 1).

1 $$ {C}_{\mathrm{brain}}={C}_{\mathrm{u},\mathrm{brain}\mathrm{ISF}}\times {V}_{\mathrm{u},\mathrm{brain}}+\frac{B_{\max}\times {C}_{\mathrm{u},\mathrm{brain}\mathrm{ISF}}}{C_{\mathrm{u},\mathrm{brain}\mathrm{ISF}}+{K}_d} $$

The relative proportion of the specific binding term in C_brain_, i.e. the target-bound fraction (f_tb_), is derived from Eq. 1 (Eq. 2) and simplifies under the conditions of C_u,brainISF_ < < K_d_ (Eq. 3).

2 $$ {f}_{\mathrm{tb}}=\frac{1}{1+\frac{V_{\mathrm{u},\mathrm{brain}}\times \left({K}_d+{C}_{\mathrm{u},\mathrm{brain}\mathrm{ISF}}\right)}{B_{\max }}} $$

3 $$ {f}_{\mathrm{tb}}=\frac{1}{1+\frac{V_{\mathrm{u},\mathrm{brain}}\times {K}_d}{B_{\max }}} $$

**Figure 2:**
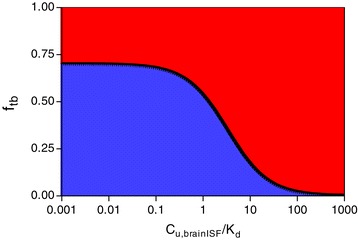
**Concentration dependence of f**_**tb**_ **for a hypothetical functioning PET tracer (solid line, Eq.** **2** **).** *Blue* and *red* areas represent the proportions of target-bound tracer and non-specific tracer in brain tissue, respectively. At low concentrations (C_u,brainISF_ < < K_d_), f_tb_ is at a plateau maximum value, which is high for functioning tracers and low for non-functioning tracers. At excessive concentrations (C_u,brainISF_ > > K_d_), the specific binding is saturated and f_tb_ negligible also for a good tracer.

It is seen from Eq. 2 that the value of f_tb_ (1) increases with increasing target density, (2) decreases with increasing non-specific binding (V_u,brain_), and (3) increases with increasing affinity to the target protein (K_d_). As illustrated in Figure 2, f_tb_ is additionally dependent on C_u,brainISF_ and has a plateau maximum value at infinitesimally low concentrations of tracer.

To facilitate comparison of *in vitro* predictions of f_tb_ and *in vivo* PET studies, a relationship (Eq. 4) was established with BP_ND_, which is essentially the ratio of B_max_ and the K_d_ × V_u,brain_ product [13].

4 $$ {f}_{\mathrm{tb}}=\frac{{\mathrm{BP}}_{\mathrm{ND}}}{{1+\mathrm{B}\mathrm{P}}_{\mathrm{ND}}} $$

While the relationships described above follows the terminology used to describe pharmacokinetics of drug transport across the blood–brain barrier and distribution within the brain tissue [14], it is consistent with our previous work using PET nomenclature [13,15]. A derivation of Eq. 1 from PET nomenclature is provided as supplementary information (Additional file 2), as is a template spreadsheet for calculation of f_tb_ (Additional file 3).

### The brain slice method

The V_u,brain_ values for all available tracers were determined using a high-throughput brain slice method exactly as described previously [11], employing tracer analysis by liquid chromatography tandem mass spectrometry (LC-MS/MS). The studies were approved by the Animal Ethics Committee of Gothenburg (234-2011).

## Results

Table 1 presents the literature data of K_d_ and B_max_ for each tracer and target along with the values of V_u,brain_ determined in rat brain slices or calculated from reported data of binding in brain homogenate (f_u,brain_). The dataset contained observations that span 3–4 orders of magnitude for each entity; the highest and lowest target expression level in the dataset was 1,407 and 0.2 nM for amyloid β protein aggregates and the nicotinic acetylcholine receptor respectively; the tracer affinities for their targets ranged from 0.016 to 270 nM for GR205171 and Remoxipride, respectively, and in terms of non-specific binding sertraline had the highest V_u,brain_ value (4,200 mL•g brain^−1^) and 2-FA-85380 the lowest (1.7 mL•g brain^−1^).

**Figure 3:**
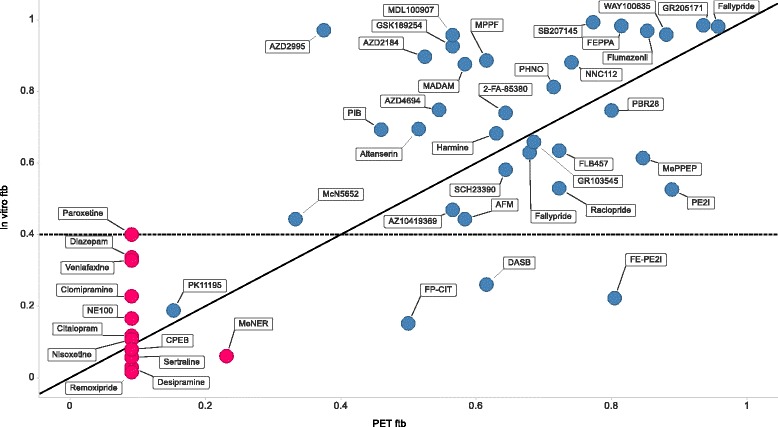
**Relationship between** ***in vitro*** **predicted and PET-derived f**_**tb**_ **for functioning tracers (** ***blue*** **) and non-functioning tracers (** ***red*** **).** The *solid* and *dashed* lines represent identity and the proposed cut-off value for f_tb_, respectively.

The target-bound fraction of tracer (f_tb_) could be derived from *in vivo* PET data for 33 of 35 functioning tracers but not for 11/12 non-functional tracers for which an arbitrary low value (0.09) was assigned (Table 1). The values of f_tb_ ranged from for very low (<0.1) for most non-functioning tracers to 0.96 for [^11^C]Fallypride. *In vitro* predictions of f_tb_, based on the brain slice method and literature data (Eq. 3), displayed a range of values from 0.02 for [^11^C]Remoxipride to 0.99 for [^11^C]Fallypride (Table 1). In general, tracers with predicted high f_tb_ values had higher observed PET values for f_tb_ than did tracers with low or moderate predicted f_tb_ values (Figure 3). A cutoff value of 0.4 for f_tb_ was used to correctly classify 31/35 functioning tracers (89% sensitivity) and 12/12 non-functioning tracers (100% specificity).

**Figure 4:**
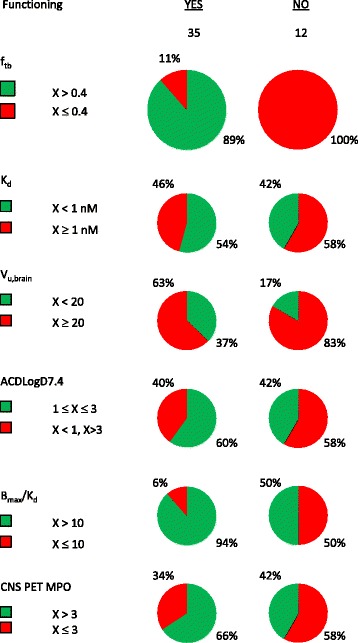
**Alignment of** ***in vitro*** **predicted f**_**tb**_ **(** ***top panel*** **) and common PET ligand selection critera with the present PET tracer dataset.**

**Figure 5:**
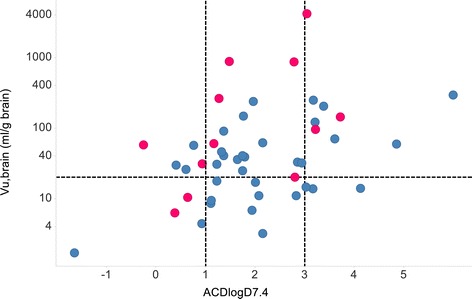
**Lack of close correlation between ACDlogD7.4 and V**_**u,brain**_ **for the functioning (** ***blue*** **) and non-functioning (** ***red*** **) PET tracers.** *Dashed* lines represent commonly applied PET-ligand selection criteria; *vertical* lines border the desired range of lipophilicity, and the *horizontal* line indicates the maximum level of non-specific binding.

Classification accuracy was determined also for the traditional CNS PET tracer selection criteria (Figure 1) and illustrated in Figure 4. Second to the presented f_tb_ classification, which correctly classified all of the non-functioning tracers, was the V_u,brain_ criterion (V_u,brain_ ≤20 mL•g brain^−1^) resulting in 10/12 correct classifications. This V_u,brain_ criterion, however, only classified 13/35 functional tracers correctly. With respect to functioning tracers, f_tb_ predictions were superseded by the B_max_/K_d_ criterion (B_max_/K_d_ ≥ 10); however, B_max_/K_d_ classified correctly only 6/12 non-functioning tracers. When combining the classification of both functional and non-functional tracers, f_tb_ prediction resulted in 43/47 (91%) correct classifications followed by the B_max_/K_d_ criterion giving 39/47 (83%) correct classifications. The MPO score, which does not rely on experimental data, made a total of 28/47 correct classifications. A poor overall rate (23/47) of correct classification was observed for the logD-based criterion, which was originally conceived with the intention to limit non-specific tissue binding while allowing a certain degree of lipophilicity to have sufficient brain exposure. To test the capability of logD to predict non-specific binding, a comparison of ACDLogD7.4 and V_u,brain_ was made and illustrated in Figure 5.

## Discussion

This study presents a mechanistically integrated approach for effective identification of PET tracer candidates based on simple and well-established theory. A prediction of the target-bound fraction of tracer (f_tb_) was made from measurements of target affinity, density, and the non-specific binding of the tracer measured in brain slices. The results show that a cutoff value of 0.4 for f_tb_ can be used to correctly classify 91% of tracer candidates as either being functioning or non-functioning. Hence, a predicted f_tb_ value greater than 0.4 can be seen as strong support to proceed with the development of a PET tracer, and a low value (<0.4) indicates small chances of success.

While keeping in mind that the aim of predicting f_tb_ is to improve decision making in the selection of PET-tracer candidates, a discussion is warranted on the agreement between predicted and observed f_tb_ at a quantitative level. The deviation from perfect agreement, which is seen as scatter around the line of identity in Figure 3, is not marginal and represents a combined ‘error’ from several sources. Obviously, the simple model used for f_tb_ (Eq. 3) may not always be sufficient to describe the full complexity of non-specific and specific binding as they occur *in vivo*. There is also considerable measurement-related error that is invariably associated with the approach taken in this study: to combine experimental data for typically three independent measurements/reports (B_max_, K_d_, and V_u,brain_) and compare with a PET-derived value of f_tb_, also carrying a measurement error. Considering that the accuracy of experimental methods such as those relating to B_max_, K_d_ and V_u,brain_ are sometimes regarded as ‘within 3-fold’; it would seem that the predictions are no worse than should be expected from experimental error alone. An illuminating example is [^11^C]DASB for which the reported K_d_ values ranged between 0.2 and 3.5 nM, corresponding to predicted f_tb_ values between 0.86 to 0.26. In this instance, the extreme value of 3.5 nM was used for K_d_ because it was the first encountered human value, despite the resulting in miss-classification as non-functioning. Another noteworthy example from this dataset is PK11195, which was misclassified by the model as non-functioning. Despite being a widely used marker for neuroinflammation, PK11195 binding in the brain has a relatively high non-specific component and was even designated as a non-functioning tracer by Zhang et al. in a recent publication [10]. In favor of the discriminating ability of the current model, the second generation TSPO radioligand PBR28 was ranked higher than PK11195. Nevertheless, PK11195 has some clinical utility, partly associated with its genotype aspecific binding, which should not be disregarded in this context.

It follows from the presented results that a default strategy at the outset of a tracer development campaign for a new target is to identify molecules with a combination of high affinity for the target and low non-specific binding, i.e. minimal values for the K_d_ × V_u,brain_ product. Depending on the density of the particular target (B_max_), different threshold values exist for K_d_ × V_u,brain_ to give rise to sufficiently high value of f_tb_ and hence a functional PET tracer. This integrative approach contrasts with the traditional process for PET tracer identification, which has been based on benchmarking against a set of discrete criteria. Integration is evidently essential as no single criterion displays prediction sensitivity and specificity that are comparable to that of the f_tb_ model. Furthermore, using all five analyzed criteria in Figure 4 as strict filters would result in the erroneous elimination of 86% of all functioning ligands; in fact, just two criteria (V_u,brain_ < 20 mL/g brain and clogD of 1–3) results in a 74% erroneous elimination. In the present dataset there is poor correlation between lipophilicity (ACDlogD7.4) and V_u,brain_ (Figure 5), suggesting that lipophilicity should not be used to predict non-specific binding. Recently, a CNS PET MPO score was developed from a PET ligand dataset [10]. This score is a composite of various calculated molecular descriptors and therefore represents an interesting integration of molecular properties that could be used alongside experimentally predicted f_tb_ or by itself to prioritize between new molecular structures before chemical synthesis is made.

A prerequisite for making *in vitro* predictions of f_tb_ is access to reliable assays for experimental determination of K_d_, V_u,brain_, and B_max_. At the stage of PET tracer development, there is almost always an assay available for the target: if not a binding assay yielding K_d_ then at least a functional assay of potency (EC_50_ or IC_50_). V_u,brain_ is best measured *in vitro* using the high-throughput brain slice methodology [11]. However, for the present integrated approach it may be sufficient to use the more readily available equilibrium dialysis brain homogenate binding assay and apply correction factors on the basis of drug pK_a_ [12]. Determination of B_max_ can pose a significant challenge since it generally requires a suitable *in vitro* radioligand. However, regardless of the B_max_ value, the initial objective of tracer optimization can be to minimize the K_d_ × V_u,brain_ product, even though the target level is not defined. If the target B_max_ is determined or known beforehand, the f_tb_ prediction model can be used not only to rank-order tracer candidates but also to assess the likelihood of being successful in identifying a tracer for a particular target. Furthermore, it is our experience that it is useful to determine B_max_ both in the preclinical species and human to facilitate the translation of f_tb_ and thereby reduce the risk of attrition.

The presented approach does not specifically address the effects of drug efflux at the blood–brain barrier or the impact of tracer metabolites in the brain, yet it predicts the present dataset with good precision and accuracy. It is possible that there is a selection bias in the dataset owing to the fact that a majority of tracers are either CNS drugs, established functioning tracers, or both. Therefore, f_tb_ predictions should be supplemented with predictions of CNS exposure using *in vivo*, *in vitro*, or *in silico* techniques. Prediction of the level of tracer metabolites in the brain is not straightforward; however, it appears to not deteriorate the predictive value of the model, which is consistent with metabolites generally having more hydrogen bond acceptors and therefore increased probability of being effluxed at the blood–brain barrier. In summary, we recognize that a poor ratio of specific to non-specific binding is one of the primary reasons for attrition in PET-tracer development and we expect this to be managed with f_tb_ predictions.

## Conclusions

A mechanistically integrated method for the identification of CNS tracer candidates was developed in which the non-specific binding, tracer affinity, and the target protein expression in the brain were taken into account. The method is rapid and accurate and is expected to facilitate the development of novel PET tracers for the molecular imaging community.

## Additional files

Additional file 1:
**CNS PET tracer dataset.**
Additional file 2:
**Derivation of target-bound fraction using PET nomenclature.**
Additional file 3:
**Tracer evaluation template. **

## Abbreviations

B_max_: target density
BP_ND_: non-displaceable binding potential
C_u,brainISF_: concentration of unbound ligand in brain interstitial fluid
f_tb_: target-bound fraction of tracer
f_u,brain_: unbound fraction in brain homogenate
K_d_: ligand affinity to target protein
MPO: multiparameter optimization
PET: positron emission tomography
V_u,brain_: unbound volume of distribution in the brain
