# Supplementary material for: Identification of positron emission tomography (PET) tracer candidates by prediction of the target-bound fraction in the brain
Source: EJNMMI Res. 2014 Sep 23;4:50. doi: 10.1186/s13550-014-0050-6 (PMC4452637; doi:10.1186/s13550-014-0050-6)
Supplement: Additional file 1: — CNS PET tracer dataset. [file 13550_2014_50_MOESM1_ESM.docx]

**European Journal of Nuclear Medicine and Molecular Imaging Research**

**Additional file 1**

**Identification of PET Tracer Candidates by Prediction of the Target Bound Fraction of Tracer in Brain**

*Markus Fridén^1,2^ Marie Wennerbe^3^, Madeleine Antonsson^3^, Maria Sandberg-Ställ^3^, Lars Farde^5^, Magnus Schou^5*^*

^1^Respiratory Inflammation and Autoimmunity Innovative Medicines, AstraZeneca R&D Mölndal, Sweden ^2^Translational PKPD, Department of Pharmaceutical Biosciences, Uppsala University, Uppsala, Sweden ^3^Cardiovascular and Metabolic Diseases Innovative Medicines, AstraZeneca R&D Mölndal Sweden ^4^CNS & Pain Innovative Medicines, AstraZeneca R&D Södertälje, Sweden ^5^AstraZeneca Translational Science Centre at Karolinska Institutet, PET Centre of Excellence, Stockholm Sweden.

^*^Author to whom correspondence should be addressed: [Magnus.Schou@astrazeneca.com](mailto:Magnus.Schou@astrazeneca.com)

**CNS PET tracer dataset**

|  |  |  | In vitro data | | | | | |  | PET data | |  | Caclulated molecular properties | | | | | | |
| --- | --- | --- | --- | --- | --- | --- | --- | --- | --- | --- | --- | --- | --- | --- | --- | --- | --- | --- | --- |
| PET tracer | Func- tioning | Target protein | Tissue | B_max_^b^ (nM) | K_d_ (nM) | f_u,brain_ | V_u,brain_^h^ | f_tb_ |  | PET f_tb_ | BP_ND_ |  | ClogP | ACD logD7.4 | PSA | MW | HBD | pKa | PET MPO |
| [^18^F]2-FA-85380 | Yes | nAChr a4b2 | Thalamus | 0.7[1] | 0.145[2] | 0.24 | 1.7 | 0.74 |  | 0.64 | 1.8[3] |  | 0.71 | -1.65 | 34 | 182 | 1 | 9.9 | 4.1 |
| [^11^C]AFM | Yes | SERT | Caudate | 38[4] | 1.04[5] | 0.06 | 46 | 0.44 |  | 0.58 | 1.4[5] |  | 3.52 | 1.32 | 11 | 276 | 0 | 8.4 | 3.9 |
| [^18^F]Altanserin | Yes | 5HT2a | Temporal cortex | 89[6]^,c^ | 0.32[7] | 0.02 | 122 | 0.70 |  | 0.51 | 1.06[8] |  | 2.58 | 3.21 | 52 | 412 | 1 | 7.8 | 3.7 |
| [^11^C]AZ10419369 | Yes | 5HT1b | Occipital cortex | 9.8[9] | 0.37[10] |  | 30 | 0.47 |  | 0.57 | 1.3[11] |  | 2.34 | 0.40 | 72 | 463 | 1 | 7.7 | 4.4 |
| [^11^C]AZD2184 | Yes | Amyloid | Cortex | 1407[12] | 4.9[13] |  | 33 | 0.90 |  | 0.52 | 1.1[14] |  | 3.42 | 2.86 | 58 | 257 | 2 | 5.0 | 3.5 |
| [^11^C]AZD2995 | Yes | Amyloid | Cortex | 1407[12] | 6.2[14] |  | 6.8 | 0.97 |  | 0.38 | 0.6[14] |  | 2.71 | 1.93 | 67 | 241 | 2 | 5.1 | 4.6 |
| [^11^C]AZD4694 | Yes | Amyloid | Cortex | 1407[12] | 2.3[15] |  | 205 | 0.75 |  | 0.55 | 1.2[16] |  | 3.38 | 3.39 | 57 | 258 | 2 | 2.3 | 3.5 |
| [^11^C]CP-126998 | Yes | AchE | Cortex | 211[17] | 0.48[18] | 0.07 | 41 | 0.92 |  |  |  |  | 3.63 | 1.75 | 44 | 390 | 0 | 8.8 | 3.5 |
| [^11^C]DASB | Yes | SERT | Caudate | 38[4] | 3.5[19] | 0.09 | 31 | 0.26 |  | 0.62 | 1.6[20] |  | 3.21 | 1.23 | 48 | 283 | 2 | 8.5 | 4.1 |
| [^18^F]Fallypride | Yes | D2 | Caudate | 26.5[21] | 0.03[22] | 0.15 | 18 | 0.98 |  | 0.96 | 22.2[23] |  | 3.18 | 1.23 | 52 | 365 | 1 | 8.1 | 4.3 |
| [^18^F]Fallypride | Yes | D2 | Thalamus | 0.9[24] | 0.03[22] | 0.15 | 18 | 0.63 |  | 0.68 | 2.11[23] |  | 3.18 | 1.23 | 52 | 365 | 1 | 8.1 | 4.3 |
| [^11^C]FE-PE2I | Yes | DAT | Striatum | 212[25] | 12[26] |  | 62 | 0.22 |  | 0.80 | 4.1[27] |  | 5.12 | 2.16 | 28 | 457 | 0 | 8.0 | 2.3 |
| [^18^F]FEPPA[iv] | Yes | TSPO | Basal ganglia | 58[28] | 0.07[29] | 0.07 | 15 | 0.98 |  | 0.81 | 4.4[30] |  | 3.65 | 3.02 | 48 | 380 | 0 | 5.4 | 3.3 |
| [^11^C]FLB457 | Yes | D2 | Thalamus | 0.9[24] | 0.02[31] | 0.11 | 26 | 0.63 |  | 0.72 | 2.6[32] |  | 3.2 | 0.60 | 52 | 371 | 1 | 9.0 | 3.9 |
| [^11^C]Flumazenil | Yes | GABA | Cortex | 71[33] | 0.7[33] |  | 3.2 | 0.97 |  | 0.85 | 5.8[34] |  | 1.29 | 2.15 | 57 | 303 | 0 | 0.9 | 5.6 |
| [^18^F]FP-CIT | Yes | DAT | Striatum | 212[25] | 33[31] |  | 36 | 0.15 |  | 0.50 | 1[32] |  | 4.42 | 1.64 | 28 | 431 | 0 | 9.3 | 2.1 |
| [^11^C]GR103545 | Yes | KOR | Striatum | 3.8[35]^,c^ | 0.048[35] | 0.07 | 41 | 0.66 |  | 0.69 | 2.18[35] |  | 4.17 | 1.36 | 49 | 414 | 0 | 8.9 | 3.3 |
| [^11^C]GR205171 | Yes | NK1 | Striatum | 55[36] | 0.016[36] | 0.05 | 57 | 0.98 |  | 0.94 | 14.5[37] |  | 3.79 | 0.76 | 78 | 432 | 2 | 9.1 | 1.7 |
| [^11^C]GSK189254A | Yes | H3 | Cortex | 8.4[38] | 0.08[38] | 0.24 | 8.5 | 0.93 |  | 0.57 | 1.3[39] |  | 3.17 | 1.11 | 54 | 351 | 1 | 9.0 | 3.9 |
| [^11^C]Harmine | Yes | MAO-A | Cerebellum | 270[40] | 5[41] | 0.04 | 25 | 0.68 |  | 0.63 | 1.7[42] |  | 3.13 | 1.75 | 33 | 212 | 1 |  | 4.8 |
| [^11^C]MADAM | Yes | SERT | Caudate | 38[4] | 0.06[43] |  | 90 | 0.88 |  | 0.58 | 1.4[44] |  | 3.59 | 1.37 | 29 | 272 | 2 | 8.5 | 2.8 |
| [^11^C]McN5652 | Yes | SERT | Caudate | 38[4] | 0.2[45] |  | 238 | 0.44 |  | 0.33 | 0.5[46] |  | 4.73 | 1.96 | 1 | 295 | 0 | 8.6 | 3.1 |
| [^11^C]MDL100907 | Yes | 5HT2a | Temporal cortex | 89[6] | 0.24[7] | 0.05 | 17 | 0.96 |  | 0.57 | 1.3[47] |  | 3.29 | 2.00 | 42 | 374 | 1 | 8.9 | 3.4 |
| [^11^C]MePPEP | Yes | CB1r | Cerebellum | 47[48] | 0.1[48] |  | 296 | 0.61 |  | 0.85 | 5.5[49] |  | 5.95 | 6.00 | 43 | 455 | 1 | 7.3 | 2.8 |
| [^18^F]MPPF | Yes | 5HT1a | Hippocampus | 350[50] | 3.3[50] | 0.1 | 14 | 0.89 |  | 0.62 | 1.6[51] |  | 3.49 | 3.17 | 41 | 435 | 0 | 6.7 | 3.1 |
| [^11^C]NNC112 | Yes | D1 | Caudate | 93[52] | 0.18[53] |  | 70 | 0.88 |  | 0.74 | 2.85[54] |  | 4.58 | 3.60 | 33 | 328 | 1 | 8.0 | 2.2 |
| [^11^C]PBR28 | Yes | TSPO | Basal ganglia | 58[28] | 1.8[55] |  | 11 | 0.75 |  | 0.80 | 3.99[56] |  | 3.4 | 2.83 | 48 | 348 | 0 | 5.4 | 3.6 |
| [^11^C]PE2I | Yes | DAT | Striatum | 212[25] | 4.9[57] |  | 39 | 0.53 |  | 0.89 | 8[58] |  | 4.87 | 1.79 | 28 | 425 | 0 | 8.1 | 2.5 |
| [^11^C]PHNO | Yes | D2/D3 | Striatum | 26.5[21]^,d^ | 0.56[59] | 0.21 | 11 | 0.81 |  | 0.71 | 2.5[60] |  | 2.5 | 2.08 | 33 | 247 | 1 | 7.6 | 4.5 |
| [^11^C]PIB | Yes | Amyloid | Cortex | 1407[12] | 2.5[12] | 0.004 | 250 | 0.69 |  | 0.46 | 0.85[61] |  | 3.99 | 3.17 | 47 | 256 | 2 | 3.0 | 3.0 |
| [^11^C]PK11195 | Yes | TSPO | Basal ganglia | 58[28] | 4.3[28] | 0.01 | 59 | 0.19 |  | 0.15 | 0.18[81] |  | 4.62 | 4.86 | 30 | 353 | 0 | 1.8 | 2.0 |
| [^18^F]Spiperone | Yes | D2 | Striatum | 26.5[21] | 0.028[62] |  | 147 | 0.87 |  |  |  |  | 2.82 | 1.76 | 53 | 396 | 1 | 8.9 | 4.2 |
| [^11^C]SB207145 | Yes | 5HT4 | Caudate | 21[63] | 0.037[11] | 0.64 | 4.4 | 0.99 |  | 0.77 | 3.4[64] |  | 2.79 | 0.92 | 74 | 341 | 2 | 8.8 | 2.9 |
| [^11^C]SCH23390 | Yes | D1 | Caudate | 93[52] | 2.1[52] | 0.06 | 32 | 0.58 |  | 0.64 | 1.8[54] |  | 4.02 | 2.94 | 24 | 288 | 1 | 8.1 | 2.6 |
| [^11^C]WAY100635 | Yes | 5HT1a | Hippocampus | 350[50] | 1.1[50] |  | 14 | 0.96 |  | 0.88 | 7.4[65] |  | 4.09 | 4.13 | 41 | 423 | 0 | 6.8 | 2.7 |
| [^11^C]Citalopram | No[66] | SERT | Caudate | 38[4] | 4.8[67] | 0.05 | 60 | 0.12 |  | 0.09 |  |  | 3.13 | 1.16 | 29 | 324 | 0 | 9.8 | 3.3 |
| [^11^C]Clomipramine | No^a^ | SERT | Caudate | 38[4] | 0.15[67] |  | 863 | 0.23 |  | 0.09 |  |  | 5.92 | 2.79 | 2 | 315 | 0 | 9.7 | 1.8 |
| [^11^C]CPEB[iv] | No[68] | ORL-1 | Whole brain | 13.5[69]^,e^ | 1.1[70] | 0.02 | 143 | 0.08 |  | 0.09 |  |  | 5.42 | 3.72 | 43 | 400 | 1 | 9.1 | 2.1 |
| [^11^C]Desipramine | No[71] | NET | Cortex | 5[72]^,c^ | 0.63[73] |  | 264 | 0.03 |  | 0.09 |  |  | 4.47 | 1.27 | 15 | 266 | 1 | 10.4 | 3.0 |
| [^11^C]Diazepam | No^a^ | GABA | Cortex | 71[33] | 7[74] |  | 20 | 0.34 |  | 0.09 |  |  | 2.96 | 2.80 | 29 | 285 | 0 | 3.4 | 4.0 |
| [^11^C]MeNER | No[75] | NET | Cortex | 5[72]^,c^ | 2.5[76] | 0.07 | 31 | 0.06 |  | 0.23 | 0.3[75] |  | 2.73 | 0.93 | 42 | 299 | 1 | 8.4 | 5.2 |
| [^11^C]NE100 | No[77] | Sigma | Whole brain | 23[78] | 1.2[77] | 0.03 | 96 | 0.17 |  | 0.09 |  |  | 5.99 | 3.22 | 19 | 356 | 0 | 9.7 | 1.0 |
| [^11^C]Nisoxetine | No[79] | NET | Cortex | 5[72] | 0.73[73] |  | 58 | 0.11 |  | 0.09 |  |  | 3.18 | -0.25 | 33 | 271 | 1 | 10.2 | 3.7 |
| [^18^F]Paroxetine | No[80] | SERT | Caudate | 38[4] | 0.065[5] |  | 876 | 0.40 |  | 0.09 |  |  | 4.24 | 1.48 | 42 | 329 | 1 | 9.7 | 3.2 |
| [^11^C]Remoxipride | No^a^ | D2 | Striatum | 26.5[21] | 270[82] |  | 6.3 | 0.02 |  | 0.09 |  |  | 3.25 | 0.38 | 52 | 371 | 1 | 9.0 | 3.9 |
| [^11^C]Sertraline | No[66] | SERT | Caudate | 38[4] | 0.15[5] |  | 4184 | 0.06 |  | 0.09 |  |  | 5.35 | 3.04 | 15 | 306 | 1 | 9.5 | 2.0 |
| [^11^C]Venlafaxine | No[80] | SERT | Caudate | 38[4] | 7.5[5] |  | 10 | 0.33 |  | 0.09 |  |  | 3.27 | 0.64 | 33 | 277 | 1 | 9.4 | 3.7 |

a Data on file

b Data refers to human brain tissue unless otherwise specified

c Monkey

d Dog

e Rat

f B_max_ value refers to caudate

g B_max_ value refers to thalamus

h Measured value in brain slices for tracers with compound material available. Calculated from reported f_u,brain_ for tracers with values of f_u,brain_ tabulated.

1. Gundisch D, Koren AO, Horti AG, Pavlova OA, Kimes AS, Mukhin AG, London ED: **In vitro characterization of 6-[18F]fluoro-A-85380, a high-affinity ligand for alpha4beta2* nicotinic acetylcholine receptors***. Synapse* 2005, **55:**89-97.

2. Chefer SI, Horti AG, Koren AO, Gundisch D, Links JM, Kurian V, Dannals RF, Mukhin AG, London ED: **2-[18F]F-A-85380: a PET radioligand for alpha4beta2 nicotinic acetylcholine receptors***. Neuroreport* 1999, **10:**2715-21.

3. Kendziorra K, Wolf H, Meyer PM, Barthel H, Hesse S, Becker GA, Luthardt J, Schildan A, Patt M, Sorger D, Seese A, Gertz HJ, Sabri O: **Decreased cerebral alpha4beta2* nicotinic acetylcholine receptor availability in patients with mild cognitive impairment and Alzheimer's disease assessed with positron emission tomography***. Eur J Nucl Med Mol Imaging* 2011, **38:**515-25.

4. Arranz B, Marcusson J: **3H]paroxetine and [3H]citalopram as markers of the human brain 5-HT uptake site: a comparison study***. J Neural Transm Gen Sect* 1994, **97:**27-40.

5. Huang Y, Zheng MQ, Gerdes JM: **Development of effective PET and SPECT imaging agents for the serotonin transporter: has a twenty-year journey reached its destination?***. Curr Top Med Chem* 2010, **10:**1499-526.

6. Lopez-Gimenez JF, Vilaro MT, Palacios JM, Mengod G: **3H]MDL 100,907 labels 5-HT2A serotonin receptors selectively in primate brain***. Neuropharmacology* 1998, **37:**1147-58.

7. Kristiansen H, Elfving B, Plenge P, Pinborg LH, Gillings N, Knudsen GM: **Binding characteristics of the 5-HT2A receptor antagonists altanserin and MDL 100907***. Synapse* 2005, **58:**249-57.

8. Fisher PM, Meltzer CC, Price JC, Coleman RL, Ziolko SK, Becker C, Moses-Kolko EL, Berga SL, Hariri AR: **Medial prefrontal cortex 5-HT(2A) density is correlated with amygdala reactivity, response habituation, and functional coupling***. Cereb Cortex* 2009, **19:**2499-507.

9. Lindhe O, Almqvist P, Kagedal M, Gustafsson SA, Bergstrom M, Nilsson D, Antoni G: **Autoradiographic Mapping of 5-HT(1B/1D) Binding Sites in the Rhesus Monkey Brain Using [carbonyl-C]zolmitriptan***. Int J Mol Imaging* 2011, **2011:**694179.

10. Maier DL, Sobotka-Briner C, Ding M, Powell ME, Jiang Q, Hill G, Heys JR, Elmore CS, Pierson ME, Mrzljak L: **N-methyl-3H3]AZ10419369 binding to the 5-HT1B receptor: in vitro characterization and in vivo receptor occupancy***. J Pharmacol Exp Ther* 2009, **330:**342-51.

11. Varnas K, Nyberg S, Halldin C, Varrone A, Takano A, Karlsson P, Andersson J, McCarthy D, Smith M, Pierson ME, Soderstrom J, Farde L: **Quantitative analysis of [11C]AZ10419369 binding to 5-HT1B receptors in human brain***. J Cereb Blood Flow Metab* 2011, **31:**113-23.

12. Klunk WE, Lopresti BJ, Ikonomovic MD, Lefterov IM, Koldamova RP, Abrahamson EE, Debnath ML, Holt DP, Huang GF, Shao L, DeKosky ST, Price JC, Mathis CA: **Binding of the positron emission tomography tracer Pittsburgh compound-B reflects the amount of amyloid-beta in Alzheimer's disease brain but not in transgenic mouse brain***. J Neurosci* 2005, **25:**10598-606.

13. Johnson AE, Jeppsson F, Sandell J, Wensbo D, Neelissen JA, Jureus A, Strom P, Norman H, Farde L, Svensson SP: **AZD2184: a radioligand for sensitive detection of beta-amyloid deposits***. J Neurochem* 2009, **108:**1177-86.

14. Forsberg A, Jureus A, Cselenyi Z, Eriksdotter M, Freund-Levi Y, Jeppsson F, Swahn BM, Sandell J, Julin P, Schou M, Andersson J, Johnstrom P, Varnas K, Halldin C, Farde L, Svensson S: **Low background and high contrast PET imaging of amyloid-beta with [11C]AZD2995 and [11C]AZD2184 in Alzheimer's disease patients***. Eur J Nucl Med Mol Imaging* 2013, **40:**580-93.

15. Jureus A, Swahn BM, Sandell J, Jeppsson F, Johnson AE, Johnstrom P, Neelissen JA, Sunnemark D, Farde L, Svensson SP: **Characterization of AZD4694, a novel fluorinated Abeta plaque neuroimaging PET radioligand***. J Neurochem* 2010, **114:**784-94.

16. Cselenyi Z, Jonhagen ME, Forsberg A, Halldin C, Julin P, Schou M, Johnstrom P, Varnas K, Svensson S, Farde L: **Clinical validation of 18F-AZD4694, an amyloid-beta-specific PET radioligand***. J Nucl Med* 2012, **53:**415-24.

17. Van Den Beukel I, Dijcks FA, Vanderheyden P, Vauquelin G, Oortgiesen M: **Differential muscarinic receptor binding of acetylcholinesterase inhibitors in rat brain, human brain and Chinese hamster ovary cells expressing human receptors***. J Pharmacol Exp Ther* 1997, **281:**1113-9.

18. Bencherif B, Endres CJ, Musachio JL, Villalobos A, Hilton J, Scheffel U, Dannals RF, Williams S, Frost JJ: **PET imaging of brain acetylcholinesterase using [11C]CP-126,998, a brain selective enzyme inhibitor***. Synapse* 2002, **45:**1-9.

19. Hummerich R, Reischl G, Ehrlichmann W, Machulla HJ, Heinz A, Schloss P: **DASB -in vitro binding characteristics on human recombinant monoamine transporters with regard to its potential as positron emission tomography (PET) tracer***. J Neurochem* 2004, **90:**1218-26.

20. Ginovart N, Wilson AA, Meyer JH, Hussey D, Houle S: **Positron emission tomography quantification of [(11)C]-DASB binding to the human serotonin transporter: modeling strategies***. J Cereb Blood Flow Metab* 2001, **21:**1342-53.

21. Tang SW, Helmeste DM, Fang H, Li M, Vu R, Bunney W,Jr, Potkin S, Jones EG: **Differential labeling of dopamine and sigma sites by [3H]nemonapride and [3H]raclopride in postmortem human brains***. Brain Res* 1997, **765:**7-12.

22. Siessmeier T, Zhou Y, Buchholz HG, Landvogt C, Vernaleken I, Piel M, Schirrmacher R, Rosch F, Schreckenberger M, Wong DF, Cumming P, Grunder G, Bartenstein P: **Parametric mapping of binding in human brain of D2 receptor ligands of different affinities***. J Nucl Med* 2005, **46:**964-72.

23. Vernaleken I, Peters L, Raptis M, Lin R, Buchholz HG, Zhou Y, Winz O, Rosch F, Bartenstein P, Wong DF, Schafer WM, Grunder G: **The applicability of SRTM in [(18)F]fallypride PET investigations: impact of scan durations***. J Cereb Blood Flow Metab* 2011, **31:**1958-66.

24. Rieck RW, Ansari MS, Whetsell WO,Jr, Deutch AY, Kessler RM: **Distribution of dopamine D2-like receptors in the human thalamus: autoradiographic and PET studies***. Neuropsychopharmacology* 2004, **29:**362-72.

25. Madras BK, Gracz LM, Fahey MA, Elmaleh D, Meltzer PC, Liang AY, Stopa EG, Babich J, Fischman AJ: **Altropane, a SPECT or PET imaging probe for dopamine neurons: III. Human dopamine transporter in postmortem normal and Parkinson's diseased brain***. Synapse* 1998, **29:**116-27.

26. Schou M, Steiger C, Varrone A, Guilloteau D, Halldin C: **Synthesis, radiolabeling and preliminary in vivo evaluation of [18F]FE-PE2I, a new probe for the dopamine transporter***. Bioorg Med Chem Lett* 2009, **19:**4843-5.

27. Sasaki T, Ito H, Kimura Y, Arakawa R, Takano H, Seki C, Kodaka F, Fujie S, Takahata K, Nogami T, Suzuki M, Fujiwara H, Takahashi H, Nakao R, Fukumura T, Varrone A, Halldin C, Nishikawa T, Suhara T: **Quantification of dopamine transporter in human brain using PET with 18F-FE-PE2I***. J Nucl Med* 2012, **53:**1065-73.

28. Venneti S, Wang G, Wiley CA: **The high affinity peripheral benzodiazepine receptor ligand DAA1106 binds to activated and infected brain macrophages in areas of synaptic degeneration: implications for PET imaging of neuroinflammation in lentiviral encephalitis***. Neurobiol Dis* 2008, **29:**232-41.

29. Wilson AA, Garcia A, Parkes J, McCormick P, Stephenson KA, Houle S, Vasdev N: **Radiosynthesis and initial evaluation of [18F]-FEPPA for PET imaging of peripheral benzodiazepine receptors***. Nucl Med Biol* 2008, **35:**305-14.

30. Rusjan PM, Wilson AA, Bloomfield PM, Vitcu I, Meyer JH, Houle S, Mizrahi R: **Quantitation of translocator protein binding in human brain with the novel radioligand [18F]-FEPPA and positron emission tomography***. J Cereb Blood Flow Metab* 2011, **31:**1807-16.

31. Halldin C, Farde L, Hogberg T, Mohell N, Hall H, Suhara T, Karlsson P, Nakashima Y, Swahn CG: **Carbon-11-FLB 457: a radioligand for extrastriatal D2 dopamine receptors***. J Nucl Med* 1995, **36:**1275-81.

32. Cervenka S, Palhagen SE, Comley RA, Panagiotidis G, Cselenyi Z, Matthews JC, Lai RY, Halldin C, Farde L: **Support for dopaminergic hypoactivity in restless legs syndrome: a PET study on D2-receptor binding***. Brain* 2006, **129:**2017-28.

33. Pandey GN, Conley RR, Pandey SC, Goel S, Roberts RC, Tamminga CA, Chute D, Smialek J: **Benzodiazepine receptors in the post-mortem brain of suicide victims and schizophrenic subjects***. Psychiatry Res* 1997, **71:**137-49.

34. Frankle WG, Cho RY, Narendran R, Mason NS, Vora S, Litschge M, Price JC, Lewis DA, Mathis CA: **Tiagabine increases [11C]flumazenil binding in cortical brain regions in healthy control subjects***. Neuropsychopharmacology* 2009, **34:**624-33.

35. Tomasi G, Nabulsi N, Zheng MQ, Weinzimmer D, Ropchan J, Blumberg L, Brown-Proctor C, Ding YS, Carson RE, Huang Y: **Determination of in vivo Bmax and Kd for 11C-GR103545, an agonist PET tracer for kappa-opioid receptors: a study in nonhuman primates***. J Nucl Med* 2013, **54:**600-8.

36. Griffante C, Carletti R, Andreetta F, Corsi M: **3H]GR205171 displays similar NK1 receptor binding profile in gerbil and human brain***. Br J Pharmacol* 2006, **148:**39-45.

37. Zamuner S, Rabiner EA, Fernandes SA, Bani M, Gunn RN, Gomeni R, Ratti E, Cunningham VJ: **A pharmacokinetic PET study of NK(1) receptor occupancy***. Eur J Nucl Med Mol Imaging* 2012, **39:**226-35.

38. Medhurst AD, Atkins AR, Beresford IJ, Brackenborough K, Briggs MA, Calver AR, Cilia J, Cluderay JE, Crook B, Davis JB, Davis RK, Davis RP, Dawson LA, Foley AG, Gartlon J, Gonzalez MI, Heslop T, Hirst WD, Jennings C, Jones DN, Lacroix LP, Martyn A, Ociepka S, Ray A, Regan CM, Roberts JC, Schogger J, Southam E, Stean TO, Trail BK, Upton N, Wadsworth G, Wald JA, White T, Witherington J, Woolley ML, Worby A, Wilson DM: **GSK189254, a novel H3 receptor antagonist that binds to histamine H3 receptors in Alzheimer's disease brain and improves cognitive performance in preclinical models***. J Pharmacol Exp Ther* 2007, **321:**1032-45.

39. Ashworth S, Rabiner EA, Gunn RN, Plisson C, Wilson AA, Comley RA, Lai RY, Gee AD, Laruelle M, Cunningham VJ: **Evaluation of 11C-GSK189254 as a novel radioligand for the H3 receptor in humans using PET***. J Nucl Med* 2010, **51:**1021-9.

40. Saura J, Kettler R, Da Prada M, Richards JG: **Quantitative enzyme radioautography with 3H-Ro 41-1049 and 3H-Ro 19-6327 in vitro: localization and abundance of MAO-A and MAO-B in rat CNS, peripheral organs, and human brain***. J Neurosci* 1992, **12:**1977-99.

41. Kim H, Sablin SO, Ramsay RR: **Inhibition of monoamine oxidase A by beta-carboline derivatives***. Arch Biochem Biophys* 1997, **337:**137-42.

42. Ginovart N, Meyer JH, Boovariwala A, Hussey D, Rabiner EA, Houle S, Wilson AA: **Positron emission tomography quantification of [11C]-harmine binding to monoamine oxidase-A in the human brain***. J Cereb Blood Flow Metab* 2006, **26:**330-44.

43. Chalon S, Tarkiainen J, Garreau L, Hall H, Emond P, Vercouillie J, Farde L, Dasse P, Varnas K, Besnard JC, Halldin C, Guilloteau D: **Pharmacological characterization of N,N-dimethyl-2-(2-amino-4-methylphenyl thio)benzylamine as a ligand of the serotonin transporter with high affinity and selectivity***. J Pharmacol Exp Ther* 2003, **304:**81-7.

44. Lundberg J, Odano I, Olsson H, Halldin C, Farde L: **Quantification of 11C-MADAM binding to the serotonin transporter in the human brain***. J Nucl Med* 2005, **46:**1505-15.

45. Elfving B, Madsen J, Knudsen GM: **Neuroimaging of the serotonin reuptake site requires high-affinity ligands***. Synapse* 2007, **61:**882-8.

46. Parsey RV, Kegeles LS, Hwang DR, Simpson N, Abi-Dargham A, Mawlawi O, Slifstein M, Van Heertum RL, Mann JJ, Laruelle M: **In vivo quantification of brain serotonin transporters in humans using [11C]McN 5652***. J Nucl Med* 2000, **41:**1465-77.

47. Meyer PT, Bhagwagar Z, Cowen PJ, Cunningham VJ, Grasby PM, Hinz R: **Simplified quantification of 5-HT2A receptors in the human brain with [11C]MDL 100,907 PET and non-invasive kinetic analyses***. Neuroimage* 2010, **50:**984-93.

48. Suter TM, Chesterfield AK, Bao C, Schaus JM, Krushinski JH, Statnick MA, Felder CC: **Pharmacological characterization of the cannabinoid CB(1) receptor PET ligand ortholog, [(3)H]MePPEP***. Eur J Pharmacol* 2010, **649:**44-50.

49. Terry GE, Hirvonen J, Liow JS, Seneca N, Tauscher JT, Schaus JM, Phebus L, Felder CC, Morse CL, Pike VW, Halldin C, Innis RB: **Biodistribution and dosimetry in humans of two inverse agonists to image cannabinoid CB1 receptors using positron emission tomography***. Eur J Nucl Med Mol Imaging* 2010, **37:**1499-506.

50. Burnet PW, Eastwood SL, Harrison PJ: **3H]WAY-100635 for 5-HT1A receptor autoradiography in human brain: a comparison with [3H]8-OH-DPAT and demonstration of increased binding in the frontal cortex in schizophrenia***. Neurochem Int* 1997, **30:**565-74.

51. Costes N, Zimmer L, Reilhac A, Lavenne F, Ryvlin P, Le Bars D: **Test-retest reproducibility of 18F-MPPF PET in healthy humans: a reliability study***. J Nucl Med* 2007, **48:**1279-88.

52. Montague DM, Lawler CP, Mailman RB, Gilmore JH: **Developmental regulation of the dopamine D1 receptor in human caudate and putamen***. Neuropsychopharmacology* 1999, **21:**641-9.

53. Andersen PH, Gronvald FC, Hohlweg R, Hansen LB, Guddal E, Braestrup C, Nielsen EB: **NNC-112, NNC-687 and NNC-756, new selective and highly potent dopamine D1 receptor antagonists***. Eur J Pharmacol* 1992, **219:**45-52.

54. Kosaka J, Takahashi H, Ito H, Takano A, Fujimura Y, Matsumoto R, Nozaki S, Yasuno F, Okubo Y, Kishimoto T, Suhara T: **Decreased binding of [11C]NNC112 and [11C]SCH23390 in patients with chronic schizophrenia***. Life Sci* 2010, **86:**814-8.

55. Owen DR, Howell OW, Tang SP, Wells LA, Bennacef I, Bergstrom M, Gunn RN, Rabiner EA, Wilkins MR, Reynolds R, Matthews PM, Parker CA: **Two binding sites for [3H]PBR28 in human brain: implications for TSPO PET imaging of neuroinflammation***. J Cereb Blood Flow Metab* 2010, **30:**1608-18.

56. Guo Q, Owen DR, Rabiner EA, Turkheimer FE, Gunn RN: **Identifying improved TSPO PET imaging probes through biomathematics: the impact of multiple TSPO binding sites in vivo***. Neuroimage* 2012, **60:**902-10.

57. Chalon S, Garreau L, Emond P, Zimmer L, Vilar MP, Besnard JC, Guilloteau D: **Pharmacological characterization of (E)-N-(3-iodoprop-2-enyl)-2beta-carbomethoxy-3beta-(4'-methylphenyl)n ortropane as a selective and potent inhibitor of the neuronal dopamine transporter***. J Pharmacol Exp Ther* 1999, **291:**648-54.

58. Odano I, Varrone A, Savic I, Ciumas C, Karlsson P, Jucaite A, Halldin C, Farde L: **Quantitative PET analyses of regional [11C]PE2I binding to the dopamine transporter--application to juvenile myoclonic epilepsy***. Neuroimage* 2012, **59:**3582-93.

59. Seeman P, Ulpian C, Larsen RD, Anderson PS: **Dopamine receptors labelled by PHNO***. Synapse* 1993, **14:**254-62.

60. Tziortzi AC, Searle GE, Tzimopoulou S, Salinas C, Beaver JD, Jenkinson M, Laruelle M, Rabiner EA, Gunn RN: **Imaging dopamine receptors in humans with [11C]-(+)-PHNO: dissection of D3 signal and anatomy***. Neuroimage* 2011, **54:**264-77.

61. Tolboom N, Yaqub M, van der Flier WM, Boellaard R, Luurtsema G, Windhorst AD, Barkhof F, Scheltens P, Lammertsma AA, van Berckel BN: **Detection of Alzheimer pathology in vivo using both 11C-PIB and 18F-FDDNP PET***. J Nucl Med* 2009, **50:**191-7.

62. Mita T, Kuno T, Nakai H, Tanaka C: **Evidence for the presence of D2 and 5-HT2 receptors in the human prefrontal cortex***. Jpn J Pharmacol* 1982, **32:**1027-32.

63. Bonaventure P, Hall H, Gommeren W, Cras P, Langlois X, Jurzak M, Leysen JE: **Mapping of serotonin 5-HT(4) receptor mRNA and ligand binding sites in the post-mortem human brain***. Synapse* 2000, **36:**35-46.

64. Marner L, Gillings N, Comley RA, Baare WF, Rabiner EA, Wilson AA, Houle S, Hasselbalch SG, Svarer C, Gunn RN, Laruelle M, Knudsen GM: **Kinetic modeling of 11C-SB207145 binding to 5-HT4 receptors in the human brain in vivo***. J Nucl Med* 2009, **50:**900-8.

65. Ito S, Suhara T, Ito H, Yasuno F, Ichimiya T, Takano A, Maehara T, Matsuura M, Okubo Y: **Changes in central 5-HT(1A) receptor binding in mesial temporal epilepsy measured by positron emission tomography with [(11)C]WAY100635***. Epilepsy Res* 2007, **73:**111-8.

66. Hume SP, Pascali C, Pike VW, Turton DR, Ahier RG, Myers R, Bateman DM, Cremer JE, Manjil LG, Dolan R: **Citalopram: labelling with carbon-11 and evaluation in rat as a potential radioligand for in vivo PET studies of 5-HT re-uptake sites***. Int J Rad Appl Instrum B* 1991, **18:**339-51.

67. Millan MJ, Dekeyne A, Papp M, La Rochelle CD, MacSweeny C, Peglion JL, Brocco M: **S33005, a novel ligand at both serotonin and norepinephrine transporters: II. Behavioral profile in comparison with venlafaxine, reboxetine, citalopram, and clomipramine***. J Pharmacol Exp Ther* 2001, **298:**581-91.

68. Ogawa M, Hatano K, Kawasumi Y, Ishiwata K, Kawamura K, Ozaki S, Ito K: **Synthesis and evaluation of 1-[(3R,4R)-1-cyclooctylmethyl-3-hydroxymethyl-4-piperidyl]-3-[11C]ethyl-1,3-dihyd ro-2H-benzimidazol-2-one as a brain ORL1 receptor imaging agent for positron emission tomography***. Nucl Med Biol* 2003, **30:**51-9.

69. Kusaka T, Yamada S, Kimura R: **Characterization of specific [3H]nociceptin binding in rat brain and spinal cord***. Biol Pharm Bull* 2001, **24:**902-5.

70. Ozaki S, Kawamoto H, Itoh Y, Miyaji M, Iwasawa Y, Ohta H: **A potent and highly selective nonpeptidyl nociceptin/orphanin FQ receptor (ORL1) antagonist: J-113397***. Eur J Pharmacol* 2000, **387:**R17-8.

71. Schou M, Sovago J, Pike VW, Gulyas B, Bogeso KP, Farde L, Halldin C: **Synthesis and positron emission tomography evaluation of three norepinephrine transporter radioligands: [C-11]desipramine, [C-11]talopram and [C-11]talsupram***. Mol Imaging Biol* 2006, **8:**1-8.

72. Mash DC, Ouyang Q, Qin Y, Pablo J: **Norepinephrine transporter immunoblotting and radioligand binding in cocaine abusers***. J Neurosci Methods* 2005, **143:**79-85.

73. Owens MJ, Morgan WN, Plott SJ, Nemeroff CB: **Neurotransmitter receptor and transporter binding profile of antidepressants and their metabolites***. J Pharmacol Exp Ther* 1997, **283:**1305-22.

74. Walker CR, Peacock JH: **Development of GABAergic function of dissociated hippocampal cultures from fetal mice***. Brain Res* 1981, **254:**541-55.

75. Logan J, Wang GJ, Telang F, Fowler JS, Alexoff D, Zabroski J, Jayne M, Hubbard B, King P, Carter P, Shea C, Xu Y, Muench L, Schlyer D, Learned-Coughlin S, Cosson V, Volkow ND, Ding YS: **Imaging the norepinephrine transporter in humans with (S,S)-[11C]O-methyl reboxetine and PET: problems and progress***. Nucl Med Biol* 2007, **34:**667-79.

76. Schou M, Zoghbi SS, Shetty HU, Shchukin E, Liow JS, Hong J, Andree BA, Gulyas B, Farde L, Innis RB, Pike VW, Halldin C: **Investigation of the metabolites of (S,S)-[(11)C]MeNER in humans, monkeys and rats***. Mol Imaging Biol* 2009, **11:**23-30.

77. Ishiwata K, Noguchi J, Ishii S, Hatano K, Ito K, Nabeshima T, Senda M: **Synthesis and preliminary evaluation of [11C]NE-100 labeled in two different positions as a PET sigma receptor ligand***. Nucl Med Biol* 1998, **25:**195-202.

78. Ishiwata K, Kobayashi T, Kawamura K, Matsuno K: **Age-related changes of the binding of [3h]SA4503 to sigma1 receptors in the rat brain***. Ann Nucl Med* 2003, **17:**73-7.

79. Haka MS, Kilbourn MR: **Synthesis and regional mouse brain distribution of [11C]nisoxetine, a norepinephrine uptake inhibitor***. Int J Rad Appl Instrum B* 1989, **16:**771-4.

80. Kamlet AS, Neumann CN, Lee E, Carlin SM, Moseley CK, Stephenson N, Hooker JM, Ritter T: **Application of palladium-mediated (18)F-fluorination to PET radiotracer development: overcoming hurdles to translation***. PLoS One* 2013, **8:**e59187.

81. Schuitemaker A, Kropholler MA, Boellaard R, van der Flier WM, Kloet RW, van der Doef TF, Knol DL, Windhorst AD, Luurtsema G, Barkhof F, Jonker C, Lammertsma AA, Scheltens P, van Berckel BN: **Microglial activation in Alzheimer's disease: an (R)-[(1)(1)C]PK11195 positron emission tomography study***. Neurobiol Aging* 2013, **34:**128-36.

82. Ross SB: **Heterogenous binding of 3H-remoxipride to membranes of rat liver and brain***. Pharmacol Toxicol* 1995, **76:**29-35.
