# Supplementary material for: Identification of positron emission tomography (PET) tracer candidates by prediction of the target-bound fraction in the brain
Source: EJNMMI Res. 2014 Sep 23;4:50. doi: 10.1186/s13550-014-0050-6 (PMC4452637; doi:10.1186/s13550-014-0050-6)
Supplement: Additional file 2: — Derivation of target-bound fraction using PET nomenclature. [file 13550_2014_50_MOESM2_ESM.docx]

**European Journal of Nuclear Medicine and Molecular Imaging Research**

**Additional file 2**

**Identification of PET Tracer Candidates by Prediction of the Target Bound Fraction of Tracer in Brain**

*Markus Fridén^1,2^ Marie Wennerbe^3^, Madeleine Antonsson^3^, Maria Sandberg-Ställ^3^, Lars Farde^5^, Magnus Schou^5*^*

^1^Respiratory Inflammation and Autoimmunity Innovative Medicines, AstraZeneca R&D Mölndal, Sweden ^2^Translational PKPD, Department of Pharmaceutical Biosciences, Uppsala University, Uppsala, Sweden ^3^Cardiovascular and Metabolic Diseases Innovative Medicines, AstraZeneca R&D Mölndal Sweden ^4^CNS & Pain Innovative Medicines, AstraZeneca R&D Södertälje, Sweden ^5^AstraZeneca Translational Science Centre at Karolinska Institutet, PET Centre of Excellence, Stockholm Sweden.

^*^Author to whom correspondence should be addressed: [Magnus.Schou@astrazeneca.com](mailto:Magnus.Schou@astrazeneca.com)

**Derivation of Target Bound Fraction using PET nomenclature**

The differential equations describing the commonly used 2-tissue compartment analysis of radiotracer binding can at approximate equilibrium conditions in vivo be related to the equation traditionally used for test tube saturation analysis [1],

$\frac{B}{F}=\frac{B_{\max}}{F+K_{d}}$ (S1)

, where B is the specific binding (pmol/g), F corresponds to free and nonspecific binding (pmol/g). K_d_ (nmol/L) and B_max_ (nmol/g brain). Eq. S1 can be rearranged to the expression

$B+F=F+\frac{B_{\max}*F}{F+K_{d}}$ (S2)

A factor f2, representing the fraction of truly free radiotracer in brain, has been introduced in the equations [2]. However, this fraction cannot be directly derived by analysis of a PET-measurement. Instead the unbound tracer concentration in the brain interstitial fluid (C_u,brainISF_, nmol/L ISF) has been introduced as an independently determined estimate.

The sum of B and F corresponds to the total concentration of radiotracer in brain tissue i.e. C_brain,_ pmol/g brain. The non-specific concentration, F, can be expressed as the product of V_u,brain_ (mL/g brain) and the unbound tracer concentration in the brain interstitial fluid (C_u,brainISF_, nmol/L ISF). By replacing the terms in Eq. S2, the following expression is obtained for the total tracer concentration in brain (Eq. S3), which is identical to Eq. 1.

$C_{brain}=C_{u,brainISF}\times V_{u,brain}+\frac{B_{\max}\times C_{u,brainISF}}{C_{u,brainISF}+K_{d}}$ (S3)

1. Farde L, Eriksson L, Blomquist G, Halldin C: **Kinetic Analysis of Central [llC]Raclopride Binding to D2-Dopamine Receptors Studied by PET-A Comparison to the Equilibrium Analysis*.*** *J Cereb Blood Flow Metab* 1989, 9:696-708.

2. Innis RB, Cunningham VJ, Delforge J, Fujita M, Gjedde A, Gunn RN, Holden J, Houle S, Huang SC, Ichise M, Iida H, Ito H, Kimura Y, Koeppe RA, Knudsen GM, Knuuti J, Lammertsma AA, Laruelle M, Logan J, Maguire RP, Mintun MA, Morris ED, Parsey R, Price JC, Slifstein M, Sossi V, Suhara T, Votaw JR, Wong DF, Carson RE: **Consensus nomenclature for in vivo imaging of reversibly binding radioligands*.*** *J Cereb Blood Flow Metab* 2007, 27:1533-9.
